# Projection of age of individuals living with HIV and time since ART initiation in 2030: estimates for France

**DOI:** 10.1002/jia2.25986

**Published:** 2022-09-29

**Authors:** Lise Marty, Yakhara Diawara, Antoine Rachas, Sophie Grabar, Dominique Costagliola, Virginie Supervie

**Affiliations:** ^1^ Sorbonne Université, INSERM Institut Pierre Louis d'Epidémiologie et de Santé Publique Paris France; ^2^ Direction de la Stratégie des Etudes et des Statistiques CNAM Paris France; ^3^ Sorbonne Université INSERM Institut Pierre Louis d'Epidémiologie et de Santé Publique AP‐HP Hôpital St Antoine Paris France; ^4^ Sorbonne Université, INSERM Institut Pierre Louis d'Epidémiologie et de Santé Publique Paris France

**Keywords:** ageing, demographic profile, time since treatment initiation, life expectancy, HIV epidemiology, modelling

## Abstract

**Introduction:**

Thanks to antiretroviral treatment (ART), people living with HIV (PLHIV) are living longer and ageing. However, ageing involves increased risks of co‐morbidities, which also depend on when PLHIV individuals started ART. To tackle the HIV age‐related upcoming challenges, knowledge of the current and future age structure of the HIV population is needed. Here, we forecast the demographic profile of the adult population living with diagnosed HIV (aPLdHIV) in France until 2030, accounting for the impact of the ART initiation period on mortality.

**Methods:**

We used national data from the French Hospital Database on HIV (ANRS CO4‐FHDH) and a sample of the National Health Data System to, first, characterize the aPLdHIV in 2018 and estimate their mortality rates according to age, sex and ART initiation period. Second, we used national HIV surveillance data to define three scenarios for the numbers of newly diagnosed HIV cases over 2019–2030: 30% decrease in HIV cases (S1), status quo situation (S2) and epidemic elimination (S3). We then combined these data using a matrix model, to project the age structure of aPLdHIV and time since ART initiation.

**Results:**

In 2018, there was an estimated 161,125 aPLdHIV (33% women), of which 55% were aged 50 or older (50+), 22% aged 60+ and 8% aged 70+. In 2030, the aPLdHIV would grow to 195,246 for S1, 207,972 for S2 and 167,221 for S3. Whatever the scenario, in 2030, the estimated median time since ART initiation would increase and age distribution would shift towards older ages: with 65–72% aPLdHIV aged 50+, 42–48% 60+ and 17–19% 70+. This corresponds to ∼83,400 aPLdHIV (28% women) aged 60+, among which ∼69% started ART more than 20 years ago (i.e. before 2010) and ∼39% ≥30 years ago (i.e. before 2000), and to ∼33,100 aPLdHIV (27% women) aged 70+, among which ∼72% started ART ≥20 years ago and ∼43% ≥30 years ago.

**Conclusions:**

By 2030, in France, close to 20% of the aPLdHIV will be aged 70+, of which >40% would have started ART more than 30 years ago. These estimates are essential to adapt co‐morbidities screening and anticipate resource provision in the aged care sector.

## 1 INTRODUCTION

Since the beginning of the HIV epidemic, great progress has been made to improve the health of people living with HIV (PLHIV). The introduction of combination antiretroviral therapy (cART) in 1996 has led to a rapid decrease in mortality [1]. With increased cART efficacy and tolerability over time, life expectancy (LE) within 3 years of cART initiation further increased and continued to increase even in the late cART era [2]. Currently, the LE of treated PLHIV approaches that of the general population [3, 4].

Consequently, HIV populations are ageing. According to UNAIDS estimates, in high‐income countries, one‐third of PLHIV were aged 50 or more in 2013 [5]. Ageing will involve new care challenges. First, age is associated with chronic conditions like non‐AIDS defining cancers, cardiovascular, renal, liver, bone and neurological diseases, and HIV infection further increases the risk of these conditions [6]. Increased burden of polypharmacy and risk of drug–drug interactions with cART could, therefore, represent an upcoming issue in HIV care [7, 8]. Second, ageing PLHIV will possibly need access to the assisted living facility for elderly people. Resource provision in the aged care sector will thus need to be addressed in the coming years, including specific HIV care training for medical staff.

To anticipate needs and resources, it is essential to foresee the number and age of PLHIV. Beyond age‐related chronic morbidities, the period at which PLHIV started antiretroviral treatment (ART) is also key, as it reflects the type of ART regimen to which individuals had been exposed, the level of immune dysfunction reached before ART initiation, which varied according to ART guidelines, and indirectly the lifetime duration with HIV. At any age, comorbidity and mortality risks are higher for individuals ageing with a longer duration of HIV infection than for individuals who are seroconverted at an older age [9]. The period of ART initiation is thus likely to influence mortality risk, but also comorbidity risk and the potential occurrence of side effects of long‐term treatment.

So far, none of the studies that projected the number and age structure of PLHIV accounted for the issue of the ART initiation period [10, 11, 12, 13, 14, 15, 16]. In this study, we propose to fill this gap and project the demographic profile of the adult population living with diagnosed HIV (aPLdHIV) in France until 2030. For this purpose, we estimated mortality rates according to the ART initiation period and considered several scenarios for the numbers of new HIV cases that will be diagnosed by 2030. We also used mortality rate estimates to provide updated estimates of LE for PLHIV currently on ART, by sex and ART initiation period and compared these estimates to those for the French general population.

## 2 METHODS

### 2.1 Data sources

Three data sources were used. First, the permanent beneficiary sample (Échantillon Généraliste des Bénéficiaires, EGB) is a representative cohort of the population covered by the main health insurance schemes, which monitors beneficiaries’ healthcare consumption and long‐term illness status. It is a sample of 1/97th of the insured individuals in France [17]. Second, the French Hospital Database on HIV (ANRS CO4‐FHDH) is a nationwide open hospital cohort created, in 1989, to enrol adult PLHIV receiving medical care, in currently 182 hospitals located throughout France [18]. The FHDH is representative of PLHIV receiving care in France [18]. Data, including demographic characteristics, biological markers and ART regimen, are collected prospectively, at each outpatient visit or hospital admission, using standardized forms. By 2019, FHDH included data on ∼210,000 individuals aged ≥18 years, including ∼106,000 with at least one follow‐up visit in 2019. Third, routine national surveillance on individuals newly diagnosed with HIV is managed by Santé Publique France [19].

### 2.2 Projecting the demographic profile of aPLdHIV

To determine the demographic profile of aPLdHIV (i.e. age, sex and ART initiation period) by 2030, we first needed data, estimates or assumptions on three parameters: (1) the demographic profile of aPLdHIV in 2018, (2) the number and age distribution of newly diagnosed HIV cases over 2019–2030 and (3) the mortality rates of aPLdHIV in 2018 and of new cases diagnosed beyond 2018. Only adults (i.e. aged ≥18 years) were included in the analysis. Specifically, we considered 14 age groups: 18–19, 20–24, 25–29, 30–34, 35–39, 40–44, 45–49, 50–54, 55–59, 60–64, 65–69, 70–74, 75–79 and ≥80.

#### 2.2.1 Demographic profile of aPLdHIV in 2018

To estimate the number and age distribution of aPLdHIV in 2018, we used data from EGB and an algorithm initially developed by the general health scheme fund to study chronic diseases (including HIV) in terms of numbers, prevalence rates, and so on [20]. For HIV, the algorithm relies on long‐term illness status, dispensations of HIV‐specific drugs and biological exams, as well as HIV diagnosis during hospital stays (see details in the Supplementary Material, Section A). We first used the algorithm to determine the numbers of beneficiaries, by sex and age group, who were receiving HIV care at least once over 2014–2018 and still alive in 2018. We then extrapolated these numbers to the whole population of France, by dividing them by the EGB representativeness (i.e. 1/97) and the proportion of the population covered by the health insurance schemes included in the EGB (i.e. 95.6% of the whole population). Then, we used data from EGB and FHDH to determine for each individual his/her date of ART initiation (Supplementary Material, Section B). We considered five ART initiation periods: 1985–1996, 1997–2005, 2006–2010, 2011–2016 and ≥2017; the choice of the periods was mainly based on the amount of available data and changes in ART eligibility criteria (Supplementary Material, Section C). Individuals who had not started ART by 2018 were assumed to initiate ART in 2019, as the median time between care entry and ART initiation was less than 1 month (FHDH data).

#### 2.2.2 New HIV cases over 2019–2030

To set the annual numbers of newly diagnosed HIV cases over 2019–2030, we projected the mean annual number of newly diagnosed cases over 2015–2018 according to three scenarios: a 30% decrease scenario (scenario 1, reference scenario), that is a linear decrease with 30% fewer cases in 2030 compared to 2015–2018, a status quo scenario (scenario 2, pessimistic scenario), with a steady annual number of cases over 2019–2030 and an epidemic elimination scenario (scenario 3, optimistic scenario), with a linear decrease in the number of cases until zero cases in 2030 (Supplementary Material, Section C, Figure S1). Scenario 1 was set as the reference as it is a broad extrapolation of the temporal trend in newly diagnosed HIV cases observed over 2012–2018. Age distribution of newly diagnosed cases over 2019–2030 was obtained by extrapolating that of cases newly diagnosed over 2010–2018 (Supplementary Material, Section C, Figure S2). We assumed that newly diagnosed individuals would initiate ART within their diagnosis year.

#### 2.2.3 Mortality rates in 2018 and beyond

To estimate mortality rates for aPLdHIV still alive in 2018, according to the ART initiation period, we used data on aPLdHIV enrolled in the FHDH who had at least one follow‐up visit between 1 January 2017 and 31 December 2019 and a known date of ART initiation.

Person‐years were calculated for each sex and ART initiation period separately. They were accumulated from 1 January 2017 or cohort enrolment, until death, loss to follow‐up (LTFU, defined as no clinical visit for 18 months, in line with French HIV guidelines [21], Supplementary Material, Section D), or 31 December 2019, whichever came first. For LTFU patients, follow‐up stopped 6 months after the last visit. Patients with a clinical visit within the 6‐month period before 31 December 2019 were censored on 31 December 2019 (Supplementary Material, Section B).

As the number of deaths is underreported in the FHDH [22], it was adjusted using data from the health insurance schemes on beneficiaries living with HIV (Supplementary Material, Section E, Tables S1 and S2).

We then used a Poisson model, with age reached in 2018 by aPLdHIV and ART initiation period as covariates, to estimate mortality rates by sex and age group, stratified by ART initiation period.

It was not possible to estimate the mortality rate for individuals who started ART in 2017 due to a lack of follow‐up data after ART initiation. Then, for these individuals and newly diagnosed cases over 2019–2030, mortality rates were assumed, conservatively, to be the same as those for individuals who started ART during 2011–2016. Likewise, due to data scarcity, the mortality rate for individuals aged 18–19 years was assumed to be the same as those for individuals aged 20–24 years.

#### 2.2.4 Projection matrix model and projection of age and time since ART initiation

We used a matrix population model to project the size of aPLdHIV until 2030, using estimates for aPLdHIV in 2018, scenarios on newly diagnosed cases over 2019–2030 and estimates of mortality rates (Supplementary Material, Section F). Distributions of time since ART initiation were also projected, together with age distributions, stratified by ART initiation period, using the same matrix model.

### 2.3 Life expectancy

Using estimated mortality rates and the life table method [23], we estimated LE for PLHIV on ART, by sex, age and ART initiation period. LE at a given age is defined as the expected number of years of life remaining for those surviving to that age (Supplementary Material, Section H). LE for the general population at ages 20, 40 and 60 years in 2018 were obtained from the Human Mortality Database (https://www.mortality.org).

### 2.4 Ethical statement

The ANRS CO4‐FHDH project was approved by CNIL (French data protection authority) on 27 November 1991, Journal Officiel, 17 January 1992. To conform to new regulations, the ANRS CO4‐FHDH was then approved by the CEREES (Expertise Committee for Research, Studies and Evaluations in the field of Health) on 20 July 2018 and as a hospital data warehouse by CNIL on 19 February 2021. The cohort received authorization to conduct research projects on the data warehouse by CNIL on 30 March 2021. All ANRS CO4‐FHDH participants signed informed consent forms mentioning the use of data for research purposes. INSERM has regulatory permanent access to EGB data, according to Article R1431‐13 of the French Public Health Code, as modified by Decree 2021–848 of 22 June 2021. All data were deidentified, thus informed consent was not necessary.

## 3 RESULTS

Demographic characteristics (age, sex and country of birth) of participants in the three data sources are provided in Table 1.

**Table 1:** Sex, age and country of birth distributions of participants to the three data sources

	EGB—2014–2018		ANRS CO4‐FHDH—2017–2019^a^		HIV surveillance—2015–2018	
	Men	Women	Men	Women	Men	Women
Total	108,871	52,253	67,721	36,321	17,141	7997
Age *N* (%)						
18–19	304 (0.3)	102 (0.2)	19 (0.0)	24 (0.1)	286 (1.7)	142 (1.8)
20–24	1522 (1.4)	1522 (2.9)	734 (1.1)	535 (1.5)	1741 (10.2)	633 (7.9)
25–29	3450 (3.2)	2638 (5.0)	2061 (3.0)	1246 (3.4)	2523 (14.7)	1231 (15.4)
30–34	5682 (5.2)	3145 (6.0)	3536 (5.2)	2587 (7.1)	2490 (14.5)	1445 (18.1)
35–39	9233 (8.5)	6189 (11.8)	4816 (7.1)	4536 (12.5)	2297 (13.4)	1384 (17.3)
40–44	9538 (8.8)	8726 (16.7)	6773 (10.0)	5832 (16.1)	2014 (11.7)	1021 (12.8)
45–49	13,190 (12.1)	7102 (13.6)	9994 (14.8)	6108 (16.8)	1936 (11.3)	681 (8.5)
50–54	21,003 (19.3)	7407 (14.2)	13,136 (19.4)	5959 (16.4)	1554 (9.1)	545 (6.8)
55–59	18,264 (16.8)	6392 (12.2)	11,504 (17.0)	4371 (12.0)	1021 (6.0)	384 (4.8)
60–64	10,451 (9.6)	3856 (7.4)	6711 (9.9)	2382 (6.6)	177 (3.6)	276 (3.4)
65–69	7102 (6.5)	1725 (3.3)	4326 (6.4)	1326 (3.7)	611 (2.3)	160 (2.0)
70–74	5682 (5.2)	1623 (3.1)	2453 (3.6)	820 (2.3)	387 (1.1)	64 (0.8)
75–79	1826 (1.7)	913 (1.7)	1060 (1.6)	363 (1.0)	65 (0.4)	21 (0.3)
80+	1623 (1.5)	913 (1.7)	598 (0.9)	232 (0.6)	31 (0.2)	10 (0.1)
Country of birth^b^ (%)						
France			48,252 (71.3)	13,304 (36.6)	10,678 (61.4)	431 (21.9)
Sub‐saharan Africa			9427 (13.9)	17,704 (48.7)	3566 (20.5)	5453 (63.9)
Europe			2969 (4.4)	933 (2.6)	870 (5.0)	247 (2.9)
America/Haïti			2778 (4.1)	2143 (5.9)	1232 (7.1)	639 (7.5)
Other (North Africa/Asia/Oceania)			4295 (6.3)	2237 (6.2)	1034 (6.01,865)	324 (3.8)

^a^ Individuals enrolled in the FHDH, who initiated ART before 2017 and had at least one follow‐up visit between 1 January 2017 and 31 December 2019.

^b^ No data on country of birth are available in the EGB. Abbreviations: EGB, permanent beneficiary sample (Échantillon Généraliste des Bénéficiaires); ANRS CO4‐FHDH, French Hospital Database on HIV.

### 3.1 Mortality rates and life expectancies

We used data on 104,042 adults (35% women), enrolled in the FHDH, who initiated ART before 2017 and had at least one follow‐up visit between 1 January 2017 and 31 December 2019 to estimate mortality rates (Supplementary Material, Figure S3), as well as LE, which are presented in Table 2 together with LE for the general population. For instance, LE for individuals aged 40 in 2018 who started ART over 2011–2016 was 39.2 years for men and 40.2 years for women. In comparison, it was, respectively, 40.9 and 46.3 years for the general population. In general, whatever the age group, LE was higher for individuals who initiated ART over 2011–2016, that is the most recent period, compared to those who initiated ART earlier. Whatever the period and age group, women had higher LE than men; however, this difference tended to decrease over time, from 7% to 10% for women who started ART over 1985–1996 to 3% to 4% for women who started ART over 2011–2016. In addition, for individuals who initiated ART over 2011–2016, that is those with the highest LE, the gap in LE compared to the general population was higher for women than for men, whatever the age group, ranging from 3.8 to 6.8 years for women and from 0.4 to 2.4 for men.

**Table 2:** Remaining life expectancy (in years) according to the age reached in 2018, for the general population^a^ and for people living with HIV (PLHIV) who initiated ART, by period of ART initiation and sex

	Age reached in 2018					
Period of ART initiation	20 years		40 years		60 years	
	Men	Women	Men	Women	Men	Women
1985–1996	x	x	35.0 (33.7–36.3)	37.4 (35.8–39.0)	19.7 (19.1–20.3)	21.6 (20.4–22.8)
1997–2005	54.4 (52.1–56.7)	56.9 (54.9–58.9)	36.2 (35.5–36.9)	38.2 (37.3–39.1)	20.5 (20.0–21.0)	22.1 (21.2–23.0)
2006–2010	56.8 (53.9–59.7)	60.0 (57.7–62.3)	38.4 (37.4–39.4)	41.0 (39.6–42.4)	22.5 (21.5–23.5)	24.4 (23.1–25.7)
2011–2016	57.7 (56.5–58.9)	59.1 (57.3–60.9)	39.2 (38.1–40.3)	40.2 (38.6–41.8)	22.9 (21.8–24.0)	23.9 (22.3–25.5)
General population^a^	60.1	65.9	40.9	46.3	23.3	27.7

Note: Mean remaining life expectancy (and 95% confidence interval) for PLHIV were estimated from mortality events among PLHIV enrolled in the French Hospital Database on HIV (ANRS CO4‐FHDH) who had at least one follow‐up clinical visit between 1 January 2017 and 31 December 2019.

^a^ Values for the general population were obtained from the Human Mortality Database (https://www.mortality.org).

### 3.2 Demographic profile of aPLdHIV in 2030

In 2018, an estimated 161,125 adults (33% women) were living with diagnosed HIV. Assuming a 30% decrease in the annual number of newly diagnosed cases over 2019–2030 (scenario 1) and using the population matrix model together with mortality rate estimates, we estimated that 195,246 adults (33% women) would be living with diagnosed HIV in 2030, that is an increase of 21% of the epidemic size. It was 207,972 assuming a steady number of newly diagnosed cases until 2030 (scenario 2) and 167,221 under the epidemic elimination scenario (scenario 3).

**Figure 1:**
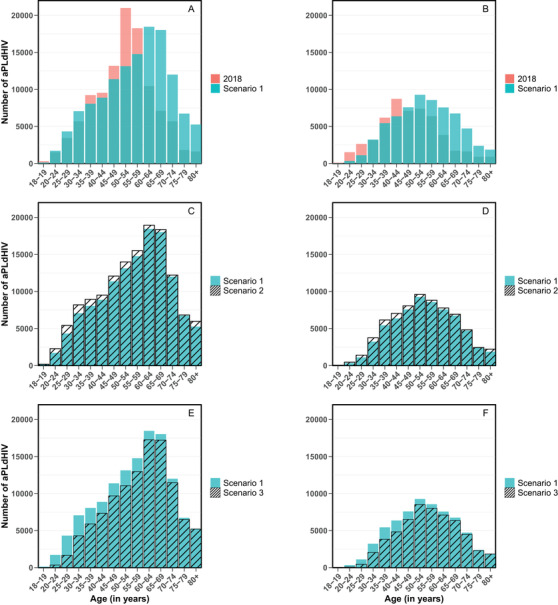
Numbers and age distributions of adults aged ≥18 years living with diagnosed HIV (aPLdHIV) in 2018 and 2030, according to different scenarios. Numbers and age distributions for men (a) and for women (b), in 2018 (in red) and in 2030 (in turquoise) for scenario 1 (i.e. 30% decrease in newly diagnosed HIV cases between 2018 and 2030). Comparison of the numbers and age distributions of adults living with diagnosed HIV in 2030 for scenario 1 (in turquoise) and scenario 2 (i.e. status quo situation with a steady annual number of new HIV cases over 2019–2030, black diagonal stripes) for men (c) and for women (d), and for scenario 1 and scenario 3 (i.e. epidemic elimination with zero new HIV cases in 2030, black diagonal stripes) for men (e) and for women (f). Detailed assumptions made for the number and age of newly diagnosed HIV cases in 2019–2030 can be found in the Supplementary Material, Section C.

For all scenarios, we found that the age distribution of aPLdHIV would shift towards older ages in 2030 (Figure 1). For scenario 1, the proportion of individuals aged ≥50 increased between 2018 and 2030, from 61% to 68% for men and from 44% to 63% for women. The proportion of individuals aged ≥60 doubled, from 24% to 47% for men and from 17% to 36% for women, like the proportion of individuals aged ≥70, from 8% to 18% for men and from 7% to 14% for women. These proportions were slightly lower for scenario 2 (Figure 1c,d) and slightly higher for scenario 3 (Figure 1e,f). Whatever the scenario, we estimated that, in 2030, there would be ∼83,400 individuals (∼28% women) aged ≥60 and ∼33,100 individuals aged ≥70 (∼27% women); in comparison, in 2018, it was, respectively, 35,715 (∼25% women) and 12,582 (∼27% women).

### 3.3 Projected time since ART initiation

**Figure 2:**
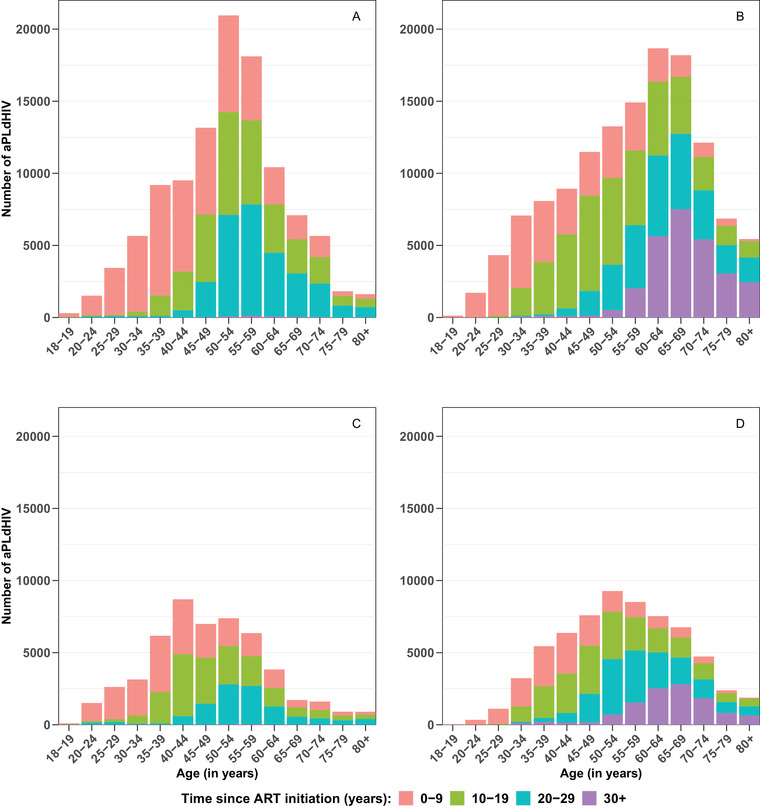
Numbers and age distributions of adults aged ≥18 years living with diagnosed HIV (aPLdHIV) in 2018 and 2030, stratified by time since ART initiation (in years): for men (a and b) and women (c and d) in 2018 (a and c) and in 2030 (b and d) for scenario 1 (i.e. 30% decrease in newly diagnosed HIV cases between 2018 and 2030). Results for other scenarios can be found in the Supplementary Material, Section I and Figures S4 and S5.

Proportions of individuals who started ART more than 20 or 30 years ago will increase over 2018–2030 (Figure 2), especially for older age groups. For brevity, we only present results for scenario 1, results for other scenarios are described in the Supplementary Material, Section I. Proportions of individuals with ≥20 years of ART exposure will increase from 27% to 42% for men and from 21% to 44% for women. In particular, for individuals aged ≥60, these proportions will increase from 43% to 68% for men and from 33% to 67% for women. Proportions of individuals with ≥30 years of ART exposure will increase from <1% to 21% for men and from <1% to 18% for women. In particular, for individuals aged ≥60, these proportions will increase from 1% to 39% for men and from <1% to 37% for women. In consequence, the median time since ART initiation will increase, especially for older age groups. For individuals aged ≥60, it will increase from 18.4 (IQR 10.4–22.4) to 25.9 years (17.6–33.4) for men and from 15.2 (7.9–21.4) to 25.8 years (16.8–33.1) for women, while for individuals aged <60, it will only increase from 9.8 (4.8–19.0) to 12.0 years (6.5–17.8) for men and from 11.0 (5.2–17.7) to 14.5 years (7.5–21.8) for women.

Of note, we estimated that, in 2030, 83,659 individuals (34% women) would have started ART ≥20 years ago and 38,492 individuals (30% women) would have started ART ≥30 years ago—versus, respectively, 40,667 and 573 in 2018 (Figure 2). Among men who started ART ≥20 years ago, 77% would be aged ≥60, 33% ≥70 and 8% ≥80. It was, respectively, 54%, 21% and 4% for women. Among men who started ART ≥30 years, 89% would be aged ≥60, 40% ≥70 and 8% ≥80. It was, respectively, 75%, 29% and 6% for women.

## 4 DISCUSSION

We projected that by 2030, the HIV epidemic in France would be growing, most likely by more than 20%, and ageing, with a doubling of the proportion of individuals aged ≥60 and ≥70. More than two‐thirds of aPLdHIV would be aged ≥50, ∼50% aged ≥60 and ∼20% aged ≥70. Interestingly, whatever the scenario considered for the epidemic dynamics over 2019–2030, we estimated that ∼83,000 individuals will be aged ≥60 in 2030, including ∼33,000 aged ≥70. Our results are in line with studies forecasting the age structure of the HIV population in other high‐income countries [10, 11, 13, 14, 16]. It was estimated that the proportion of PLHIV on ART aged ≥50 would be 73% in 2030 in the Netherlands [13] and 54% in the United States [16], and ∼75% in 2035 in the United States and Italy [14]. Bretaña et al. [10] performed projections for Australia, considering three scenarios for the future number of newly diagnosed cases over 2018–2027. They highlighted that, whatever the scenario, the age distribution of PLHIV would have its highest peak in the 55–59 age group in 2027, which aligns with our findings of highest peak in the 60–64 age group for men and 50–54 for women.

In addition, our study predicts that in 2030, in France, there will be more than 38,000 individuals who would have started ART more than 30 years ago (i.e. before 2000), with most of them being aged ≥60 (85%, ∼33,000), 37% aged ≥70 (∼14,000) and 8% aged ≥80 (∼3000). These individuals were thus exposed to the first generation of nucleoside reverse transcriptase inhibitors (AZT and D4T) and protease inhibitors, which have been associated with body morphology changes and cardiovascular diseases [24, 25]. In addition, ART duration and time living with diagnosed HIV infection have been associated with increased risk of multimorbidity [7, 9], but also with psychological morbidity and lower quality of life [26, 27], which should be considered as part of integrated HIV care. To the best of our knowledge, our study is the first to project time spent since ART initiation for an HIV population. However, previous studies emphasized other important aspects of the projected demographic profile of HIV populations, which we were unable to take into account. First, a study investigating the capacity of current cART to offer long‐term HIV control found that the median time until exhaustion of treatment options was 45.5 years (IQR 34.0–61.0 years) [28]. Furthermore, some studies showed important heterogeneity in the projections of PLHIV according to race/ethnicity, with an older projected population of white PLHIV compared to Black and Hispanic minorities [11, 29]. Other studies [13, 14] focused on the burden and prevalence of age‐related co‐morbidities: Smit et al. [13] predicted that in 2030, 84% of PLHIV in the Netherlands will have at least one age‐related non‐communicable disease, with 28% having three or more, mainly due to cardiovascular disease. This could generate complications due to drug–drug interactions for 40% of patients with the currently recommended first‐line HIV regimen. Finally, a study for Australia [15] highlighted that the number of PLHIV in non‐metropolitan areas, where the PLHIV median age is higher, is expected to increase at a greater rate than that in the major cities.

We also found that the LE of adults who started ART from 2011 onwards was either equal to or approaching that of the general population: for instance, at age 60, in 2018, it was ∼23 and ∼24 years for, respectively, men and women living with diagnosed HIV versus, respectively, ∼23 and ∼28 years in the general population. Individuals who started ART in 2005 or earlier, and are still alive in 2018, had lower LE, but the difference was only 2–4 years. This can have important implications for health‐related insurance policies for PLHIV. Marcus et al. [30] reported an overall LE of 56.0 years at age 21 over 2014–2016, close to our estimates for individuals of age 20 in 2018, ranging from 51.4 to 57.4 years. Studies that estimated LE for earlier periods of follow‐up found, expectedly, lower LE than ours [3, 31]. We also found that although women living with diagnosed HIV had higher LE than men, the gap in LE compared to the general population was higher for women than for men, which is in line with previous results [2, 3]. Potential explanations for this higher gap include later access to HIV care for women than for men. However, in France, the time between infection and care entry was estimated to be shorter for women than for men [32, 33]. Another explanation is that among women living with diagnosed HIV in France, a vast majority were born abroad (63%, of which 77% in sub‐Saharan African countries, Table 1), while among men, a vast majority were born in France (71%). Hence, differences in socio‐economic levels and access to healthcare system between born‐abroad and born‐in‐France individuals, but also the stigma and marginalization, probably play an important role in the observed sex difference in the LE gap between PLHIV and the general population [34, 35].

The main novelty of our approach is that it accounts for the impact of the ART initiation period on mortality rates to project the demographic profile of the HIV population. In addition, our projections for the population size aged ≥60 are robust to assumptions regarding epidemic dynamics over 2019–2020. However, our study also has a number of limitations. First, the projection method and LE estimates rely on the assumption that age‐specific mortality rates estimated over 2017–2019 will remain constant over 2019–2030. On one hand, lower mortality beyond 2019 would lead to higher LE estimates and a larger HIV population in 2030. On the other hand, higher mortality among older age groups, due to covid‐19 during 2020–2021 for instance, could lead to a decrease in LE, total population size and proportions of older PLHIV in 2030. Second, several limitations affect HIV care data. Data on deaths in FHDH were not comprehensive and were adjusted for under‐reporting, with potential inaccurate adjustments (Supplementary Material, Section G for details). As health insurance schemes do not collect data on the HIV exposure group, this factor could not be accounted for. Third, we could not include individuals aged <18 years for population size estimates. According to health insurance and HIV surveillance data, this could represent ∼5200 individuals in 2030, comprising ∼4000 individuals aged <18 years living with diagnosed HIV in 2018, plus ∼100 individuals who could be newly diagnosed each year over 2019–2030. Fourth, our global LE estimates do not capture the comorbidity‐free LE. This was estimated to remain much lower for PLHIV than for the general population (9.5 years difference in a US cohort of insured adults [30]). Finally, with HIV becoming more prevalent among older adults, transmission risk in higher age groups might increase, if, for instance, older PLHIV are not adherent to their treatment. This may impact the age distribution of individuals becoming newly infected, with for instance more individuals seroconverting at an older age. As a consequence, interventions explicitly targeting older individuals may be needed, as older individuals were recently shown to be at increased risk of delayed presentation for HIV care [36].

## 5 CONCLUSIONS

By 2030, in France, close to 20% of the adult population living with diagnosed HIV will be aged ≥70 (i.e. ∼33,000 individuals), of which >40% would have started ART more than 30 years. Ageing of the HIV population has important implications for care, generating an increase in comorbidity prevalence and treatment complexity. Our findings can help to measure the burden of ageing and anticipate healthcare needs, resource provision and screening guidelines in HIV care, in France but also in other high‐income countries. Indeed, our estimates probably provide a broad picture of what is likely to occur in terms of HIV population ageing in other settings, with similar historical access to ART and free access to care.

## COMPETING INTERESTS

VS reports lecture fees from ViiV (2019), Gilead (2019, 2020) and Janssen‐Cilag (2020), outside the submitted work.

DC reports an HIV grant from Janssen (2019–2020) and personal fees from Gilead (2020) and Pfizer (2022) for lectures, outside the submitted work.

LM, AR, SG and YD declare no competing interests.

## AUTHORS’ CONTRIBUTIONS

LM and VS designed the research. LM performed the research. All authors analysed the data. LM and VS drafted the manuscript. All authors critically revised the manuscript for important intellectual content.

## FUNDING

FHDH is funded by the ANRS‐MIE, the Institut National de la Santé et de la Recherche Médicale (INSERM) and the French Ministry of Health.

## Supporting information

Supplementary Material
**Figure S1**. Annual number of newly diagnosed HIV cases over 2019–2030 according to the three scenarios, for men (A) and for women (B). In green: linear decrease with 30% fewer cases in 2030 compared to 2015–2018 (scenario 1). In blue: status quo situation with a steady annual number of new HIV cases over 2019–2030 (scenario 2). In red: epidemic elimination with zero new HIV cases in 2030 (scenario 3).
**Figure S2**. Projected adult newly diagnosed HIV cases by age group at diagnosis over 2019–2030 for men (A) and for women (B).
**Figure S3**. Mortality rates of adults aged ≥20 years living with diagnosed HIV in 2018, according to age and ART initiation period, for men (A) and women (B).
**Figure S4**. Numbers and age distributions of adults aged ≥18 years living with diagnosed HIV (aPLdHIV) in 2018 and 2030, stratified by time since ART initiation (in years): for men (A and B) and women (C and D) in 2018 (A and C) and in 2030 (B and D) under scenario 2 (i.e. status quo situation with a steady annual number of new HIV cases over 2019–2030).
**Figure S5**. Numbers and age distributions of adults aged ≥18 years living with diagnosed HIV (aPLdHIV) in 2018 and 2030, stratified by time since ART initiation (in years): for men (A and B) and women (C and D) in 2018 (A and C) and in 2030 (B and D) under scenario 3 (i.e. epidemic elimination with zero new HIV cases in 2030).
**Table S1**. Data from the French National Health Data System and the FHDH for the years 2017, 2018 and 2019 on the number of individuals living with diagnosed HIV and deaths among them, stratified by age group.
**Table S2**. Data from the French National Health Data System for the years 2017, 2018 and 2019 on the number of beneficiaries living with diagnosed HIV (BLHIV) and deaths among them, stratified by age group.

## Data Availability

Anyone can submit a research project to the ANRS CO4‐FHDH scientific committee and obtain access to the data after approval by the scientific committee. Applicants should use a standardized form available on the ANRS CO4‐FHDH website (https://anrs‐co4.fhdh.fr) to describe the context and objectives of the study. The scientific committee reviews the submitted projects twice a year. For successful applicants with adequate statistical expertise, the data can be transferred with French data protection agency CNIL approval; otherwise, the ANRS CO4 FHDH statistical centre analyses the data cooperatively with the applicant. Our institution has permanent access to the EGB given by its governance (ministerial steering). Without permanent access, an access request to the EGB for a project requires authorization from the Health Data Hub (https://www.health‐data‐hub.fr/depot). Anyone can submit a research project to Santé Publique France and obtain access to the routine HIV surveillance data after approval by the scientific committee, by writing to ANSP‐DMI‐VIC@santepubliquefrance.fr.
