# Supplementary material for: Projection of age of individuals living with HIV and time since ART initiation in 2030: estimates for France
Source: J Int AIDS Soc. 2022 Sep 29;25(Suppl 4):e25986. doi: 10.1002/jia2.25986 (PMC9523002; doi:10.1002/jia2.25986)

# Supplementary Material

**Projection of age of individuals living with HIV and time since ART initiation in 2030: estimates for France**

Lise Marty^1^, Yakhara Diawara^1^, Antoine Rachas^2^, Sophie Grabar^3^, Dominique Costagliola^4^, Virginie Supervie^1^

^1^ Sorbonne Université, INSERM, Institut Pierre Louis d’Epidémiologie et de Santé Publique, F75012, Paris, France

^2^ Direction de la Stratégie, des Etudes et des Statistiques, CNAM, F-75000 Paris, France

^3^ Sorbonne Université, INSERM, Institut Pierre Louis d’Epidémiologie et de Santé Publique, AP-HP, Hôpital St Antoine, F75012, Paris, France

^4^ Sorbonne Université, INSERM, Institut Pierre Louis d’Epidémiologie et de Santé Publique, F75013, Paris, France

1. **Identification of individuals living with diagnosed HIV in 2018 from the EGB**

Adapting a method initially developed by the general health scheme fund to study several chronic diseases (including HIV) [1], in terms of numbers, prevalence rates, etc., we used the following criteria to estimate, from a representative sample of the national health data system (Échantillon Généraliste des Bénéficiaires, EGB), the number of individuals living with diagnosed HIV in 2018:

- Persons with long-term illness status (ALD, qualifying for 100% reimbursement of medical expenses), with ICD 10 code for HIV, in any year over 2014-2018, and still alive in 2018;
- and/or persons hospitalized in medicine, surgery, obstetrics, with HIV as the main or associated diagnosis (i.e. with ICD 10 code for HIV) at admission, in any year over 2014-2018, and still alive in 2018;
- and/or persons hospitalized in medicine, surgery, obstetrics with HIV infection as complication or associated morbidity (main, related, or associated diagnosis from the hospital discharge database) in any year over 2014-2018, and still alive in 2018;
- and/or persons with at least three dispensations of HIV-specific treatment at different point in time within one year, in any year over 2014-2018, and still alive in 2018 (excluding Truvada dual therapy and Hepatitis B specific treatments);
- and/or persons with at least one HIV-specific medical biology exam (including antiretroviral genotypic resistance tests, antiretroviral plasma concentration measurement, viral load quantification) in any year over 2014-2018;

We applied this set of criteria over the year 2014-2018, in order to identify all individuals living with diagnosed HIV, whether engaged in care or not in 2018. Indeed, applying this set of criteria only for the year 2018 would have only allowed identifying individuals living with diagnosed HIV if they were engaged in HIV care and/or have been hospitalized in 2018, whereas individuals who had interrupted their follow-up and did not have any hospitalizations in 2018 would have remained unidentified.

Age distribution was obtained by considering the age reached by the beneficiaries in 2018.

1. **Extracting the date of ART initiation of individuals living with diagnosed HIV in 2018**

We used data from the EGB and the FHDH cohort to determine for each individual living with diagnosed HIV in 2018 his/her date of ART initiation. These two data sources complement each other as follows. On one hand, information on the date of ART initiation in EGB is not complete (and less complete than in the FHDH cohort), since EGB was created in 2005, with retrospective care consumption data from 2004 only, while FHDH data are available since 1985. On the other hand, as with any patient cohort, FHDH collects information on individuals who are engaged in care but it loses track of patients who interrupt HIV care, whereas EGB includes all patients, whether they are engaged in care or not.

Therefore, individuals living with diagnosed HIV in 2018 were split into two groups, according to whether they were engaged in care in 2018 or not. For individuals who were engaged in care in 2018, we extracted the date of ART initiation from FHDH, and defined five periods of ART initiation (1985-1996, 1997-2005, 2006-2010, 2011-2016 and ≥2017). For individuals who were not engaged in care in 2018, we extracted the date of ART initiation from EGB, and defined only four periods of ART initiation, because of shorter data collection period (<2006, 2006-2010, 2011-2016 and ≥2017). For both groups, we assumed that individuals who had not initiated ART in 2018 would do so in 2019.

To identify the two groups (engaged in care in 2018 or not), we proceeded as follows. In a first step, we applied the set of criteria described in section A over the years 2014-2018, and as a second step, we applied the same set of criteria but only for the year 2018. Individuals identified with the first step correspond to the whole population of individuals living with diagnosed HIV in 2018 (engaged in care in 2018 or not) while individuals identified with the second step only correspond to those engaged in care in 2018. Then, from individuals identified in the first step but not in the second step, we determined those who were not engaged in care in 2018.

The choice of the periods was based on the amount of data available and changes in ART eligibility criteria. Treat-all strategy was adopted at the end of 2013 in France, but due to lack of follow-up data we did not consider a group of patients who initiated ART over 2014-2016. The two other milestones in terms of treatment guidelines that we considered are the following ones:

- 1997: advent of cART;
- 2010: change in CD4 count eligibility from 350 to 500;

Since the period from 1997 to 2010 was large, and overall mortality was steadily decreasing throughout this period, we split it into two, 1997-2005 and 2006-2010, in order to provide more precise mortality estimates, as the amount of data allowed it. All together we thus consider four periods: before 1997, 1997-2005 and 2006-2010 and 2011-2016. Beyond 2016, we assumed that mortality rates would be the same as the mortality rates for individuals who had started ART during 2011-2016.

1. **Number and age of newly diagnosed HIV cases over 2019-2030**

The projected mean annual numbers of new HIV cases diagnosed over 2019-2030 according to the three scenarios are presented in Figure S1: a 30% decrease in the annual number of cases by 2030 (scenario 1), a status quo scenario, with a steady annual number of cases over 2019-2030 (scenario 2), and an epidemic elimination scenario (scenario 3), with a linear decrease in the number of cases up to zero case in 2030.

To set age distributions of cases over 2019-2030, we first performed, for each sex, a linear regression of the annual numbers of newly diagnosed HIV cases over 2010-2018 against time, with age as a categorical covariable. Estimated regression coefficients were then used to project, by sex, age distributions of new cases from 2019 to 2030. Using these age distributions (figure S2) and the scenario-dependent annual numbers of new cases over 2019-2030 (figure S1), we then obtained the annual numbers of new HIV diagnoses by age group over 2019-2030.

1. **Definition of patients lost to follow-up based on French HIV guidelines**

We chose an 18-month period to define patients lost to follow-up because French HIV guidelines have moved towards fewer laboratory tests and medical visits to health care centers per year [2]. Specifically, the 2013 French guidelines recommend administration of ART to each PLHIV whatever the CD4 count; a once-a-year visit to an infectious disease specialist for patients on ART whose HIV infection has been under control (undetectable viral load and CD4 T cell count >500 cells/mm3) for one year without comorbidity; and a clinical and immuno-virological check-up at least every 6 months, which can be performed by the primary care physician.

1. **Correction of the reported number of death cases**

The numbers of deaths reported in the FHDH over 2017-2019 (Table S1) were adjusted for under-reporting, using data from the health insurance schemes on beneficiaries living with diagnosed HIV (BLHIV); personal communication from A. Rachas. To do so, we first estimated the coverage of FHDH, i.e. the proportion of all individuals living with diagnosed HIV in France who are enrolled in FHDH. Second, we estimated the total number of death cases among individuals living with diagnosed HIV in France. We then used these two estimates to obtain the correction factor to adjust for under-reporting of deaths in FHDH.

The coverage of FHDH for each age group $a$ and year $t$, noted $\rho_{a,t}$, was estimated using the ratio of the number of individuals living with diagnosed HIV enrolled in FHDH database over the total number of BLHIV from all health insurance schemes, for each year and age group (Table S1):

$\rho_{a,t}=\frac{N_{a,t}^{F}}{N_{a,t}}$ (1)

where $N_{a,t}^{F}$ is the number of individuals living with diagnosed HIV with at least one medical visit registered in the FHDH and $N_{a,t}$ the total number of BLHIV from all insurance schemes (Table S1);

The total number of death cases among individuals living with diagnosed HIV in France for each year $t$ and age group $a$, noted $D_{a,t}$, had to be estimated using data on deaths among BLHIV from the main health insurance scheme, also called the general Social Security scheme, because data on deaths are not complete for the other insurance schemes. Specifically, we extrapolated the number of deaths reported among BLHIV of the main health insurance scheme to all insurance schemes using the ratio of the number of BLHIV of the general Social Security scheme over the total number of BLHIV from all insurance schemes, for each year and age group:

$D_{a,t}=D_{a,t}^{'}\frac{N_{a,t}^{'}}{N_{a,t}}$ (2)

with $D_{a,t}^{'}$ the number of deaths among BLHIV of the general Social Security scheme, $N_{a,t}^{'}$ the number of BLHIV of the general Social Security scheme and $N_{a,t}$, as aforementioned, the total number of BLHIV from all insurance schemes (Table S2).

$\rho_{a,t}$, and $D_{a,t},$obtained from equations (1) and (2), were then used to estimate a factor to correct unreported deaths for each age group $a$ and year $t$, noted $\delta_{a,t}$, using the following formula :

$\delta_{a,t}=\rho_{a,t} \frac{D_{a,t}}{D_{a,t}^{F}}$ (3)

where $D_{a,t}^{F}$ is the number of deaths reported in FHDH database for each age group $a$ and year $t$ (Table S1).

Each death case in FHDH database were then multiplied by the factor $\delta_{a,t}$, obtained from equation (3), to adjust for under-reporting of deaths.

1. **Age-structured projection matrix model**

The projection method was adapted from a Leslie matrix model for age-structured population. Each year, the population was stratified by age and period of ART initiation. We divided age into 63 age groups, i.e. one group for each age over ages 18-79 years and one group for the last open-ended age group, ≥80 years, and the period of ART initiation into five periods (i.e. 1985-1996, 1997-2005, 2006-2010, 2011-2016 and ≥2017).

From 2019 to 2030, the number of individuals living with diagnosed HIV in age group $a$ in year $t$, $N_{a,t}$, corresponds to (i) the sum over all ART initiation periods of individuals of age $a-1$ living with diagnosed HIV in year $t-1$with ART initiation period $k$ who aged one year and thus survived up to year $t$, plus (ii) the number of new HIV cases of age $a$ diagnosed in year $t$, who initiated ART within the same year of their diagnosis, $D_{a,t,k}$:

$N_{a,t}=\sum_{k=1}^{5} N_{a-1,t-1,k}s_{a-1,k}+D_{a,t,k}$, (4)

with $s_{a-1,k}$ the probability of surviving from age $a-1$ to age $a$, for individuals who started ART in time period $k$, which is defined as:

$s_{a-1,k}=e^{-m_{a-1,k}}$,

where $m_{a-1,k}$ is the estimated mortality rate of individuals of age$a-1$ who initiated treatment during the time period $k$.

Equation (4) was thus iterated 12 times (from 2019 to 2030) to obtain the number and age distribution of aPLdHIV in 2030.

Then the total count of individuals living with diagnosed HIV in each year $t$ was obtained by summing the count of individuals living with diagnosed HIV over all age groups $a$ and over all ART initiation periods $k$.

1. **Sources of uncertainty in the data and method**

Calculation steps presented in this study involve different data sources or estimates, and each of them involves a certain degree of uncertainty.

First, EGB is a sample, covering 1/97^th^ of the insured persons in France. However, it is a representative sample, which allows us to make extrapolations to obtain the total number of people identified as HIV-positive.

Second, number of deaths had to be adjusted for under-reporting: overall, we estimated that under-reporting of death events was 37%. To adjust the number of deaths, we used data on death events among people identified as HIV-positive who were insured in the general health insurance scheme, as data on deaths are only exhaustive for this scheme. Data from the general health insurance scheme are the most exhaustive data source in France, as individuals are tracked until deaths or migration, which is a strength. Nevertheless, two sources of uncertainty can arise in this process. First, the algorithm used to identify HIV-positive persons is probably not perfect, although it is the reference to estimate HIV prevalence in France. Second, we had to extrapolate the number of deaths observed among individuals insured with the general health insurance scheme to obtain to the number of deaths among all people identified as HIV-positive, not only those insured with the general health insurance scheme. The general health insurance scheme includes 84% of people identified as HIV-positive in all health insurance schemes, the uncertainty in this extrapolation is therefore quite low.

Third, uncertainty lies in the number of newly diagnosed HIV cases that will be observed in the future. This source of uncertainty has been taken into account by considering several scenarios (see aforementioned answer).

1. **Life Expectancy**

Using estimated mortality rates and life table method [3], we estimated life expectancy for PLHIV on ART in France, by sex, age and ART initiation period. For the oldest open-ended age group (≥80), data on follow-up care and deaths were scarce. Therefore, following other studies [4,5], we obtained an estimate of the mortality rate for individual aged ≥80 by multiplying the French general population mortality rate for individuals aged ≥80 by an average standardized mortality ratio (SMR), representing the ratio between PLHIV mortality rate and general population mortality rate. The average SMR was obtained by first calculating SMRs for age groups 70-74 and 75-79 and then by averaging these two values.

1. **Projected time since ART initiation : results from scenarios 2 and 3**
   1. **Scenario 2 : status quo situation with a steady annual number of new HIV cases over 2019-2030**

We found that the proportions of individuals who started ART more than 20 and 30 years ago will increase over 2018-2030 (Figure S4), especially for older age groups. The overall proportions of individuals who started ART ≥20 years (respectively, ≥30 years) will increase from 27% to 39% (respectively, from <1% to 19%) for men, and from 21% to 42% (respectively, from <1% to 17%) for women. In particular, among those aged ≥60, these proportions will increase from 43% to 67 % (respectively, from 1% to 38 %) for men, and from 33% to 66% (respectively, from <1% to 36%) for women. In consequence, median time since ART initiation will increase, especially for older age groups. In particular, for individuals aged ≥60, it will increase from 18.4 (interquartile range (IQR) 10.4 – 22.4) to 25.5 years (IQR 17.0 – 33.3) for men, and from 15.2 (IQR 7.9-21.4) to 25.3 years (IQR 15.8 – 32.9) for women, while for those aged <60, it will only increase from 9.8 (IQR 4.8 – 19.0) to 10.5 years (IQR 5.5 – 17.3) for men, and from 11.0 (IQR 5.2-17.7) to 13.3 years (IQR 6.5 – 21.3) for women.

Of note, we estimated that, in 2030, 83,659 individuals (34% women) would have started ART ≥20 years ago, i.e. before 2010, including 38,492 individuals (30% women) that would have started ART ≥30 years ago - versus respectively 40,667 and 573 in 2018 – with the vast majority of them being aged ≥60 (Figure 2). For those who would have started ART ≥20 years ago, for men, 77% would be aged ≥60, 33% ≥70, 8% ≥80, and for women, 54% would be ≥60, 21% ≥70, 4% ≥80, while for those who would have started ART ≥30 years ago, for men, 89% would be aged ≥60, 40% ≥70, 8% ≥80, and for women, 75% being aged ≥60, 29% ≥70, 6% ≥80.

- 1. **Scenario 3 : epidemic elimination with zero new HIV cases in 2030**

We found that proportions of individuals who started ART more than 20 and 30 years ago will increase over 2018-2030 (Figure S5), especially for older age groups. The overall proportions of individuals who started ART more than 20 years (respectively, ≥30 years) will increase from 27% to 49% (respectively, from <1% to 24%) for men, and from 21% to 51% (respectively, from <1% to 20%) for women. In particular, among those aged ≥60, these proportions will increase from 43% to 72% (respectively, from 1% to 41%) for men, and from 33% to 71% (respectively, from <1% to 39%) for women. In consequence, median time since ART initiation will increase, especially for older age groups. In particular, for individuals aged ≥60, it will increase from 18.4 (interquartile range (IQR) 10.4 – 22.4) to 26.8 years (IQR 18.9 – 33.5) for men, and from 15.2 (IQR 7.9-21.4) to 26.6 years (IQR 18.4 – 33.3) for women, while for those aged <60, it will only increase from 9.8 (IQR 4.8 – 19.0) to 14.8 years (IQR 10.5 – 19.6) for men, and from 11.0 (IQR 5.2-17.7) to 16.9 years (IQR 11.5 – 23.5) for women.

Of note, we estimated that, in 2030, 83,659 individuals (34% women) would have started ART ≥20 years ago, i.e. before 2010, including 38,492 individuals (30% women) that would have started ART ≥30 years ago - versus respectively 40,667 and 573 in 2018 – with the vast majority of them being aged ≥60 (Figure 2). For those who would have started ART ≥20 years ago, for men, 77% would be aged ≥60, 33% ≥70, 8% ≥80, and for women, 54% would be ≥60, 21% ≥70, 4% ≥80, while for those who would have started ART ≥30 years ago, for men, 89% would be aged ≥60, 40% ≥70, 8% ≥80, and for women, 75% being aged ≥60, 29% ≥70, 6% ≥80.

**References**

1. Sécurité Sociale - l’Assurance maladie. Cartographie des pathologies et des dépenses de l’Assurance Maladie [Internet]. Available from: https://assurance-maladie.ameli.fr/etudes-et-donnees/par-theme/pathologies/cartographie-assurance-maladie

2. Fournier AL, Yazdanpanah Y, Verdon R, Lariven S, Mackoumbou-Nkouka C, Phung BC, et al. Incidence of and risk factors for medical care interruption in people living with HIV in recent years. PLoS One. 2019;14(3):e0213526.

3. Chiang CL. The life table and its construction. In: Introduction to stochastic processes in Biostatistics. New York: John Wiley and Sons; 1968. p. 198–214.

4. Antiretroviral Therapy Cohort Collaboration. Life expectancy of individuals on combination antiretroviral therapy in high-income countries: a collaborative analysis of 14 cohort studies. Lancet. 2008;372:293–9.

5. Antiretroviral Therapy Cohort Collaboration. Survival of HIV-positive patients starting antiretroviral therapy between 1996 and 2013: a collaborative analysis of cohort studies. Lancet HIV. 2017;4(8):e349–56.

**Table S1. Data from the French National Health Data System and the FHDH for the years 2017, 2018 and 2019 on the number of individuals living with diagnosed HIV and deaths among them, stratified by age group.**

| **Year** $\boldsymbol{(t)}$ | **Age group***$\boldsymbol{(a)}$ | **Number of individuals living with HIV with at least one medical visit registered in the FHDH, (**$\boldsymbol{N}_{\boldsymbol{a,t}}^{\mathbf{F}}$**)** | **Number of BLHIV from all health insurance schemes, (**$\boldsymbol{N}_{\boldsymbol{a,t}}\boldsymbol{)}$ | **Coverage of the FHDH, (**$\boldsymbol{\rho}_{\boldsymbol{a,t}}$**)**** | **Number of deaths reported in the FHDH, (**$\boldsymbol{D}_{\boldsymbol{a}\boldsymbol{,t}}^{\mathbf{F}}$**)** |
| --- | --- | --- | --- | --- | --- |
| 2017 | 18-34 | 13250 | 17087 | 77.5% | 12 |
| 2017 | 35-54 | 57521 | 78277 | 73.5% | 282 |
| 2017 | 55-64 | 22928 | 32422 | 70.7% | 232 |
| 2017 | 65-74 | 8152 | 11803 | 69.1% | 124 |
| 2017 | ≥75 | 1898 | 3307 | 57.4% | 72 |
| 2018 | 18-34 | 13410 | 17090 | 78.5% | 26 |
| 2018 | 35-54 | 57563 | 76502 | 75.2% | 254 |
| 2018 | 55-64 | 25570 | 35611 | 71.8% | 249 |
| 2018 | 65-74 | 9158 | 12857 | 71.2% | 121 |
| 2018 | ≥75 | 2203 | 3713 | 59.3% | 88 |
| 2019 | 18-34 | 11854 | 17750 | 66.8% | 15 |
| 2019 | 35-54 | 54362 | 74871 | 72.6% | 205 |
| 2019 | 55-64 | 27310 | 39022 | 70.0% | 233 |
| 2019 | 65-74 | 9747 | 14014 | 69.6% | 144 |
| 2019 | ≥75 | 2494 | 4186 | 59.6% | 84 |

BLHIV: beneficiaries living with diagnosed HIV ;

*five age groups (18-34, 35-54, 55-64, 65-74, 75+) to comply with available statistics from the French National Health Data System.

** estimated as $\rho_{a,t}=\frac{N_{a,t}^{F}}{N_{a,t}}$.

**Table S2. Data from the French National Health Data System for the years 2017, 2018 and 2019 on the number of beneficiaries living with diagnosed HIV (BLHIV) and deaths among them, stratified by age group.**

| **Year** $\left( t \right)$ | **Age group***$(a)$ | **Number of BLHIV from all health insurance schemes****, ($N_{a,t})$ | **Number of BLHIV of the general Social Security scheme,**  ($N_{a,t}^{'})$ | **Number of deaths among BLHIV of the general Social Security** **scheme,** ($D_{a,t}^{'})$ | **Estimated number of deaths among BLHIV from all health insurance schemes*****, ($D_{a,t})$ |
| --- | --- | --- | --- | --- | --- |
| 2017 | 18-34 | 17087 | 14087 | 18 | 22 |
| 2017 | 35-54 | 78277 | 66482 | 451 | 531 |
| 2017 | 55-64 | 32422 | 26392 | 443 | 544 |
| 2017 | 65-74 | 11803 | 9388 | 202 | 254 |
| 2017 | ≥75 | 3307 | 2599 | 169 | 215 |
| 2018 | 18-34 | 17090 | 13871 | 22 | 27 |
| 2018 | 35-54 | 76502 | 65083 | 440 | 517 |
| 2018 | 55-64 | 35611 | 29446 | 451 | 545 |
| 2018 | 65-74 | 12857 | 10460 | 220 | 270 |
| 2018 | ≥75 | 3713 | 2968 | 169 | 211 |
| 2019 | 18-34 | 17750 | 15038 | 26 | 31 |
| 2019 | 35-54 | 74871 | 64752 | 336 | 389 |
| 2019 | 55-64 | 39022 | 33138 | 472 | 556 |
| 2019 | 65-74 | 14014 | 11770 | 289 | 344 |
| 2019 | ≥75 | 4186 | 3447 | 198 | 240 |

*five age groups (18-34, 35-54, 55-64, 65-74, 75+) to comply with available statistics from the French National Health Data System.

** includes data from the general Social Security scheme as well as all other schemes;

*** estimated as $D_{a,t}=D_{a,t}^{'}\frac{N_{a,t}^{'}}{N_{a,t}}$

**Figure S1. Annual number of newly diagnosed HIV cases over 2019-2030 according to the three scenarios,** for men (A) and for women (B). In green: linear decrease with 30% fewer cases in 2030 compared to 2015-2018 (scenario 1). In blue: status quo situation with a steady annual number of new HIV cases over 2019-2030 (scenario 2). In red: epidemic elimination with zero new HIV cases in 2030 (scenario 3).

**Figure S2. Projected adult newly diagnosed HIV cases by age group at diagnosis over 2019-2030 for men (A) and for women (B).**

**Figure S3. Mortality rates of adults aged ≥20 years living with diagnosed HIV in 2018, according to age and ART initiation period, for men (A) and women (B).**

**Figure S4. Numbers and age distributions of adults aged ≥18 years living with diagnosed HIV (aPLdHIV) in 2018 and 2030, stratified by time since ART initiation (in years):** for men (A and B) and women (C and D) in 2018 (A and C) and in 2030 (B and D) under scenario 2 (i.e. status quo situation with a steady annual number of new HIV cases over 2019-2030).


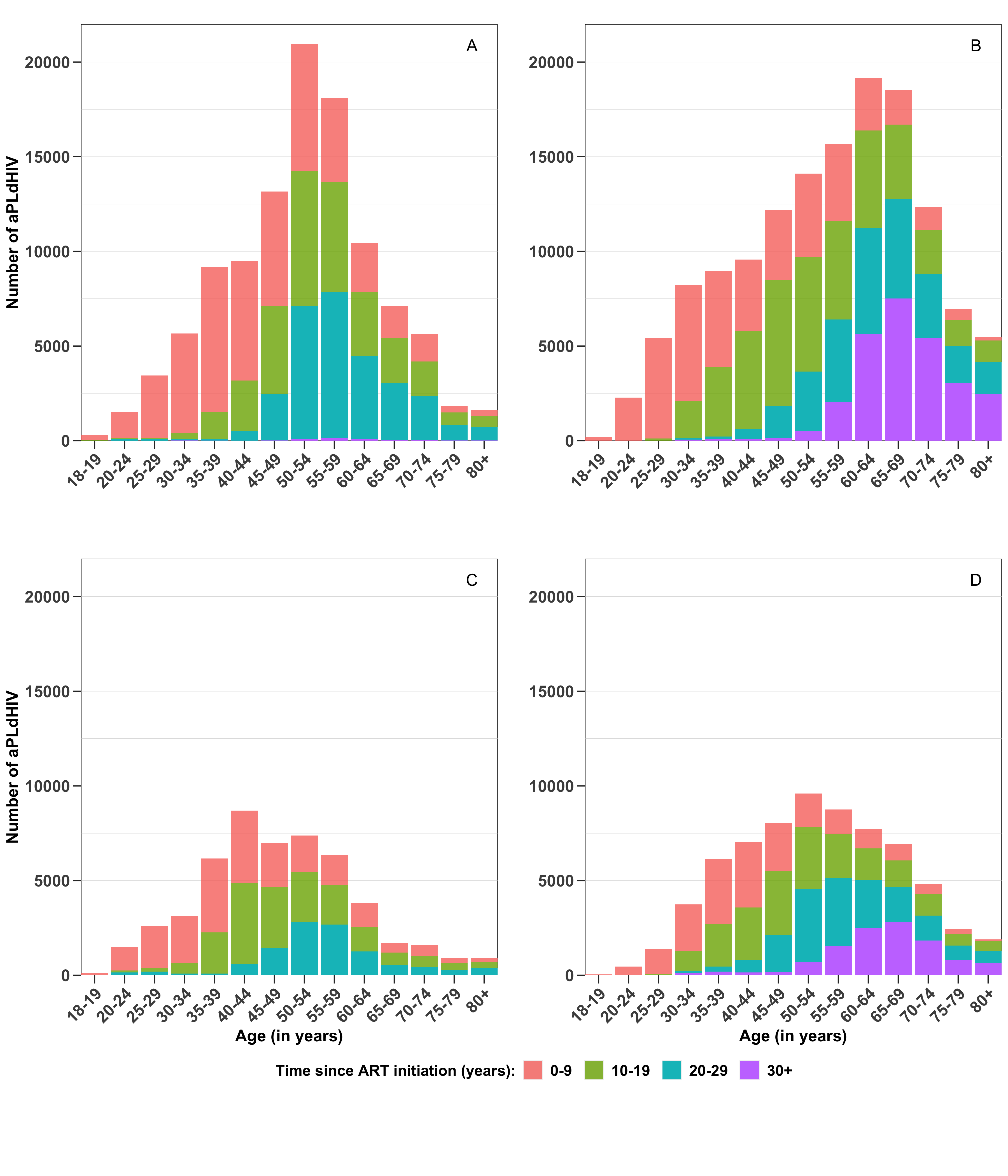


**Figure S5. Numbers and age distributions of adults aged ≥18 years living with diagnosed HIV (aPLdHIV) in 2018 and 2030, stratified by time since ART initiation (in years):** for men (A and B) and women (C and D) in 2018 (A and C) and in 2030 (B and D) under scenario 3 (i.e. epidemic elimination with zero new HIV cases in 2030).


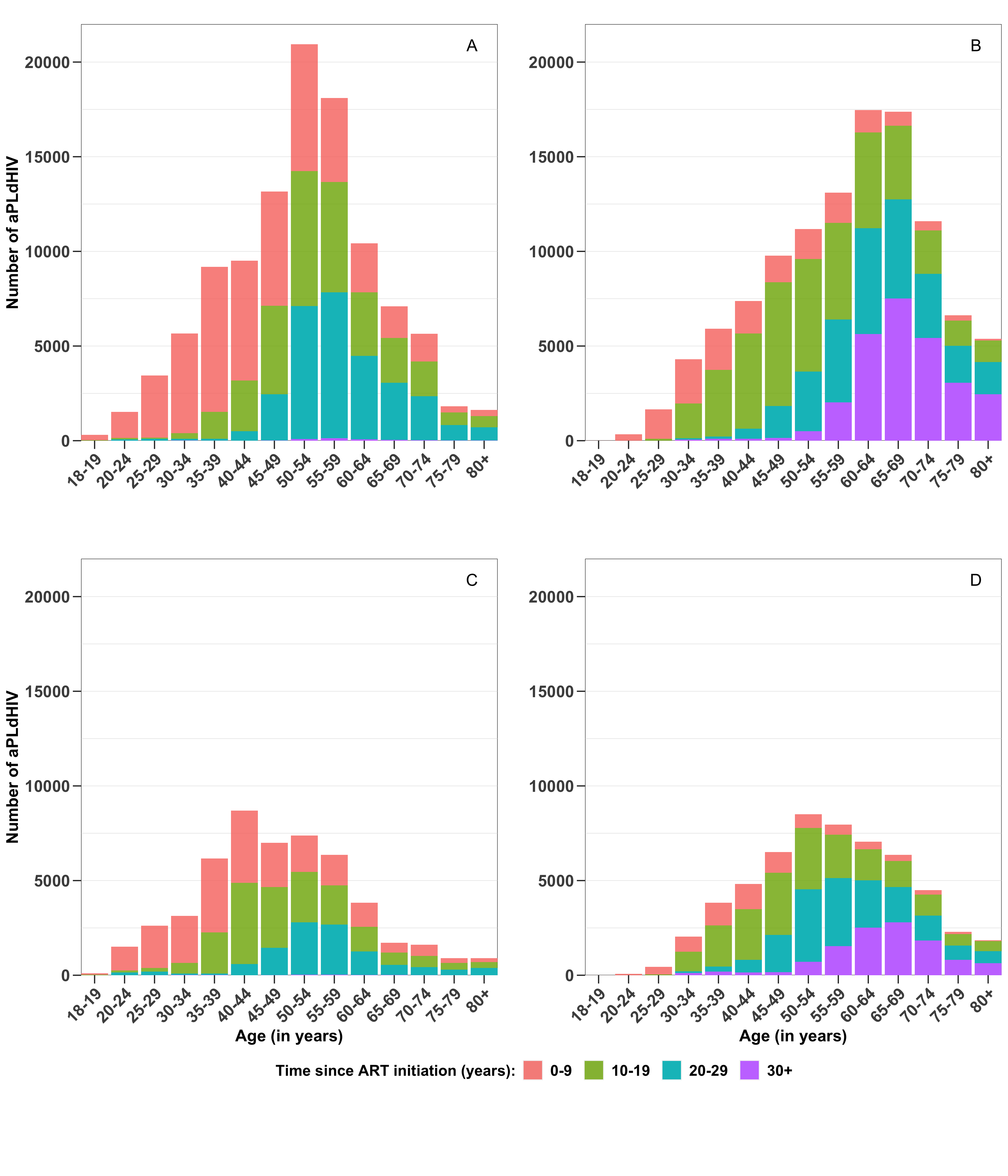

Supplement: Supplementary file 1 — Supplementary Material Figure S1. Annual number of newly diagnosed HIV cases over 2019–2030 according to the three scenarios, for men (A) and for women (B). In green: linear decrease with 30% fewer cases in 2030 compared to 2015–2018 (scenario 1). In blue: status quo situation with a steady annual number of new HIV cases over 2019–2030 (scenario 2). In red: epidemic elimination with zero new HIV cases in 2030 (scenario 3). Figure S2. Projected adult newly diagnosed HIV cases by age group at diagnosis over 2019–2030 for men (A) and for women (B). Figure S3. Mortality rates of adults aged ≥20 years living with diagnosed HIV in 2018, according to age and ART initiation period, for men (A) and women (B). Figure S4. Numbers and age distributions of adults aged ≥18 years living with diagnosed HIV (aPLdHIV) in 2018 and 2030, stratified by time since ART initiation (in years): for men (A and B) and women (C and D) in 2018 (A and C) and in 2030 (B and D) under scenario 2 (i.e. status quo situation with a steady annual number of new HIV cases over 2019–2030). Figure S5. Numbers and age distributions of adults aged ≥18 years living with diagnosed HIV (aPLdHIV) in 2018 and 2030, stratified by time since ART initiation (in years): for men (A and B) and women (C and D) in 2018 (A and C) and in 2030 (B and D) under scenario 3 (i.e. epidemic elimination with zero new HIV cases in 2030). Table S1. Data from the French National Health Data System and the FHDH for the years 2017, 2018 and 2019 on the number of individuals living with diagnosed HIV and deaths among them, stratified by age group. Table S2. Data from the French National Health Data System for the years 2017, 2018 and 2019 on the number of beneficiaries living with diagnosed HIV (BLHIV) and deaths among them, stratified by age group. [file JIA2-25-e25986-s001.docx]
